# Case report: Olverembatinib monotherapy: the chemotherapy-free regimen for an elderly patient with relapsed Ph-positive acute lymphoblastic leukemia

**DOI:** 10.3389/fphar.2023.1320641

**Published:** 2023-11-16

**Authors:** Jianghua Ding, Wen Li

**Affiliations:** Department of Hematology and Oncology, Jiujiang University Affiliated Hospital, Jiujiang, Jiangxi, China

**Keywords:** olverembatinib, elderly, philadelphia chromosome, B-cell, acute lymphoblastic leukemia

## Abstract

**Background:** The advent of first- and second-generation BCR/ABL1 tyrosine kinase inhibitors (TKIs), such as imatinib and dasatinib, has markedly improved the clinical outcomes of patients with philadelphia chromosome–positive acute lymphoblastic leukemia (Ph^+^-ALL). However, due to acquired drug resistance, most Ph^+^-ALL patients experience relapse. Thus, third-generation BCR/ABL1 TKIs, including ponatinib and olverembatinib, have been developed with the aim of overcoming drug resistance.

**Case report**: A 79-year-old woman presented with intermittent fever and fatigue for 4 days. After comprehensive cytogenetic examination, the patient was diagnosed with Ph^+^-B-ALL. Starting on 22 September 2021, a combined regimen of flumatinib and vincristine/prednisone (VP) was administered for seven cycles, followed by flumatinib maintenance therapy. The patient remained in first complete molecular remission (1^st^ CMR) for 19 months. On 12 March 2023, she again complained of fatigue and loss of appetite for nearly a month. A comprehensive examination showed Ph^+^-B-ALL relapse with additional E255V mutation, although T315I mutation was negative. In view of her frail physical condition, she received olverembatinib monotherapy and achieved second CMR (second CMR). No severe toxicities were recorded except for mild fatigue. At present, she has been in second CMR for over 6 months.

**Conclusion:** For elderly patients with relapsed Ph^+^-ALL, olverembatinib monotherapy may offer a novel option with a good safety profile, suggesting the feasibility of a chemo-free regimen.

## Introduction

Prior to the advent of BCR/ABL1 tyrosine kinase inhibitors (TKIs), philadelphia chromosome (Ph)–positive acute lymphoblastic leukemia (Ph^+^ ALL) was associated with a dismal prognosis (Jabbour et al., 2023). At the time, the primary treatment strategy was intense induction chemotherapy followed by allogeneic stem cell transplantation (allo-SCT) at first complete remission (CR). The long-term survival rate was approximately 10%–35% (Foa and Chiaretti, 2022). However, since the revolutionary incorporation of BCR/ABL1 TKIs into chemotherapy, the clinical outcomes of Ph^+^ ALL patients have been dramatically improved, with a long-term survival rate ranging from 40% to 65% for first- and second-generation TKIs such as imatinib, dasatinib, and nilotinib (Haddad et al., 2023). Nonetheless, relapses still occur, in particular in patients who do not receive consolidative allo-SCT. Up to 75% of relapsed patients harbor the T315I mutation in which threonine at amino acid position 315 (in the ABL sequence) is replaced with isoleucine (Wang et al., 2022). Thus, addressing the secondary T315I mutation remains a serious medical problem in the clinical setting.

In recent years, novel agents targeting the T315I mutation have been developed. Ponatinib is the first third-generation TKI that has more potent activity on BCR-ABL1 tyrosine kinase than other TKIs and can overcome T315I mutations (Kidoguchi et al., 2021). The U.S. Food and Drug Administration (FDA) approved ponatinib as a second-line treatment for patients carrying relapsed and refractory chronic myeloid leukemia (CML) and Ph^+^ ALL with T315I mutation. However, ponatinib is not currently available in China. Olverembatinib, a third-generation BCR/ABL1 TKI developed by Ascentage Pharma in China, was approved as a standard treatment for CML with T315I mutation on November 24, 2021. Recently, there were a few case reports on olverembatinib in newly diagnosed or relapsed Ph^+^ ALL patients (Liu et al., 2023; Tan et al., 2023). Inspired by this, we applied olverembatinib in an elderly relapsed Ph^+^ ALL patient with the additional chromosomal abnormality of an E255V mutation. Encouragingly, the patient achieved molecular complete remission.

## Case report

A 79-year-old woman complained of intermittent fever and fatigue for 4 days and was admitted to the hematology wards of our hospital on 21 September 2021. Her body temperature fluctuated between 38.5°C and 39.2°C, which was accompanied by chills and loss of appetite. She presented with chest tightness, but without cough, expectoration, or hemoptysis symptoms. No mucosal bleeding symptoms, such as easy bruising, epistaxis, and gingival bleeding, were recorded.

The patient had a significant medical history of hypertension for over 20 years and coronary heart disease and chronic atrial fibrillation for 4 years. In 2019, she received percutaneous transluminal coronary angioplasty (PTCA) with stent implantation treatment. She had regularly taken rivaroxaban, metoprolol, rosuvastatin, and benazepril for maintenance treatment.

On 21 September 2021, the patient’s routine blood test showed that her total leukocyte count was 12.25 (G/L), her neutrophil count was 0.88 (G/L), and her lymphocyte count was 11.26 (G/L). The hemoglobin and platelet counts were 87 (g/L) and 20 (G/L), respectively. Bone marrow (BM) cytology revealed ALL with 96.5% leukemic blast cells. The immunophenotype was detected by flow cytometry as CD10^−^, CD19-and CD22-positive B-ALL. Chromosome karyotype and genetic testing revealed [46, XX], BCR/ABL1 (P190) ^+^, WT1^+^, and IKZF1 mutation. The patient was finally diagnosed with Ph^+^-B-ALL.

On 27 September 2021, combined induction therapy consisted of flumatinib (600 mg/day), vincristine (1.5 mg/m^2^ with 2 mg maximum dose once weekly, on days 1, 8, 15, 22) and prednisone (60 mg daily, on days 1–28) for one cycle of 28 days. After the therapy, she achieved complete molecular remission (1st-CMR). Subsequently, she received consolidative treatment of flumatinib plus VP regimens with the same dose as induction therapy for seven cycles from 1 November 2021 to 15 July 2022. She was then treated with maintenance therapy of flumatinib (600 mg/day) alone for eleven cycles (30 days of every cycle) from 16 July 2022 to 11 March 2023 and remained in CMR. During this period, the patient experienced two severe infections with fungi and Gram-negative bacteria and recovered after antibiotic therapy. For central nervous system leukemia (CNS-L) prophylaxis, she was administered 13 intrathecal injections (IT) of 10 mg methotrexate, 50 mg cytosine arabinoside and 5 mg dexamethasone each time, from November 2021 to July 2022, i.e., twice every month for the first 3 months and then once a month for next 8 months. No abnormality was detected in cerebrospinal fluid (CSF).

**FIGURE 1:**
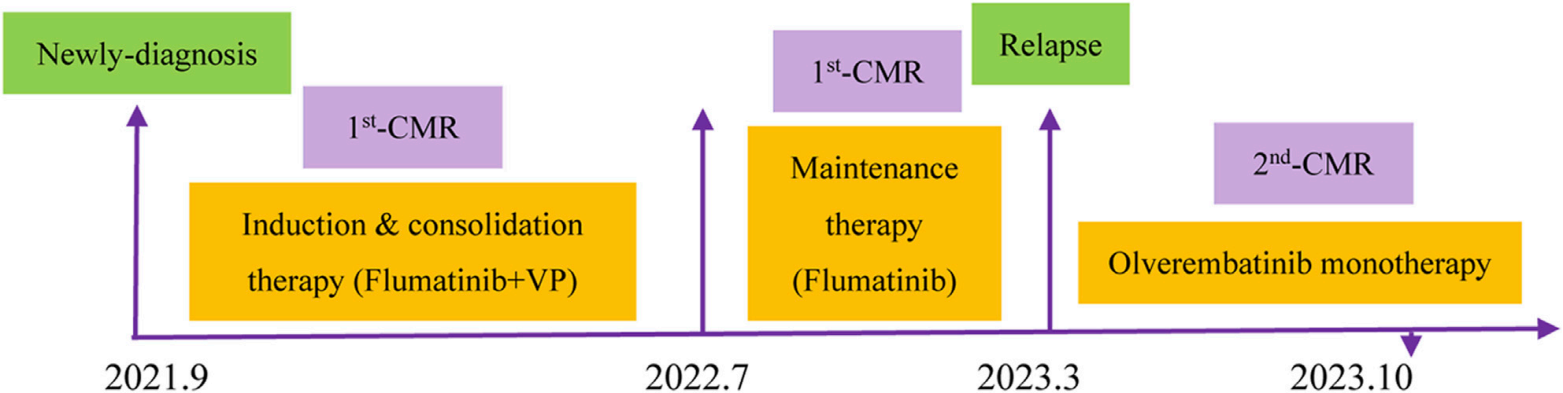
Detailed time course of the patient’s clinical course and therapeutic regimen.

On March 12, 2023, she presented with the chief complaint of fatigue and loss of appetite for nearly a month and was then re-admitted to our hospital. A physical examination showed sternum tenderness. The blood routine examination revealed a total leukocyte count of 4.53×10^9^/L, a neutrophil count of 1.7×10^9^/L, a lymphocyte count of 2.75×10^9^/L, a hemoglobin count of 88 g/L, and a platelet count of 54×10^9^/L. Bone marrow aspiration cytology found 84% ALL-blast cells. The chromosome karyotype was [46, XX]. The genetic detection was positive for BCR/ABL1 (33.89%) and E255V mutation, but negative for T315I mutation. The patient was diagnosed with relapsed Ph^+^-ALL with BCR/ABL1 and secondary E255V mutation. In consideration of her age and frail physical condition, the patient was treated with oral olverembatinib (40 mg/day, qod) alone for one cycle of 30 days according to the package information from 21 March 2023. Encouragingly, she again achieved CMR (second-CMR) after two cycles of olverembatinib monotherapy. Furthermore, no severe toxicities were observed except for mild fatigue. The patient has remained in CMR for over 6 months. On 15 September 2023, the blood routine examination revealed a total leukocyte count of 8.01×10^9^/L, a neutrophil count of 6.55×10^9^/L, a lymphocyte count of 1.01×10^9^/L, a hemoglobin count of 138 g/L, and a platelet count of 226×10^9^/L. During the period, the patient received only twice of IT on June 23, and 24 July 2023. No abnormal cells were observed in CSF. Now, she is still under routine follow-up. The course about treatment and progression were seen in Figure 1.

## Discussion

Histologically, Ph^+^-ALL has been deemed as a high-risk acute leukemia subtype that has a very poor prognosis due to chemotherapeutic resistance. Over the past two decades, dramatic progress has been made in the therapeutic landscape of Ph^+^-ALL. At present, incorporating BCR/ABL1-TKIs into cytotoxic chemotherapy has become the standard of care for Ph^+^-ALL patients.

As a first-generation TKI, imatinib plus intensive chemotherapy was evaluated in adult patients with Ph^+^-ALL in a UKALLXII/ECOG E2993 clinical study. The results showed that 43.2% of the patients (*n* = 1909) relapsed, and 91.3% of the patients experienced relapse within 3 years. The 4-year overall survival (OS) was 38%. The cumulative risk of 3- and 5-year relapse was 40% and 43%, respectively (Ganzel et al., 2020). Dasatinib, a second-generation TKI, exhibited better clinical outcomes in comparison with imatinib, i.e., the 4-year event-free survival (EFS) and 4-year OS increased from 48.9% to 69.2%, respectively, for imatinib to 71% and 88.4%, respectively, for dasatinib (Shen et al., 2020). However, both imatinib and dasatinib resulted in a mere 20%–50% complete molecular remission (CMR), and most resistant patients harbor the T315I mutation (Haddad et al., 2023). Thus, third-generation BCR/ABL1 TKIs have emerged with the aim of overcoming the T315I mutation.

Ponatinib (Takeda, Japan), the first third-generation TKI, is the pan-BCR/ABL1 agent against T315I-resistant ABL1 mutation. Accumulative studies revealed that ponatinib-based combination therapeutic strategies have been employed in relapsed and refractory Ph^+^-ALL patients with previous first- or second-BCR/ABL1 TKI treatment. Wang H et al. treated 19 patients with relapsed and refractory Ph^+^-ALL with the venetoclax, ponatinib, and dexamethasone (VPD) regimen. The CMR rate was 42.1% (8/19) (Wang et al., 2022). Thomas C et al. retrospectively analyzed the results of ponatinib plus blinatumomab (CD3/CD19 bispecific antibody) in 26 relapsed and refractory Ph^+^-ALL patients. Of these patients, 88.5% (23/26) achieved CMR, and the mOS and EFS were 20 and 15.3 months, respectively (Couturier et al., 2021). Additionally, ponatinib alone may show superior survival and a shorter time to achieve CMR compared to dasatinib alone for Ph^+^-ALL with CNS-L patients with or without the T315I mutation (Zhu et al., 2023). Currently, an increasing number of studies have begun to explore the role of ponatinib as a first-line treatment for Ph^+^-ALL. Notably, the CMR percentage and 3-year OS rate were greater in the combined ponatinib plus chemotherapy group than those in the first- or second-generation BCR/ABL1 TKI plus chemotherapy group (79% vs. 34%, *p* = 0.034; 79% vs. 50%, *p* = 0.05) (Jabbour et al., 2018). These results strongly support the potential of first- and second-line ponatinib treatment in Ph^+^-ALL. Unfortunately, ponatinib has not yet been brought to market in China.

Olverembatinib, a domestic third-generation BCR/ABL1 TKI, was designed to effectively target pan-BCR/ABL1 mutations, including T315I, indicating a promise for Ph^+^-ALL. Recently, only a few reports have begun to investigate the clinical effect of olverembatinib in relapsed Ph^+^-ALL patients. In the study by Liu et al., olverembatinib plus glucocorticoid, VP, or hyper CAVD were used to treat five adult relapsed Ph^+^-ALL patients. All of them achieved CR, including two patients who achieved CMR and three who achieved major molecular remission (MMR). The EFS and OS ranged from 4 to 9 months and 10–92 months, respectively. One patient harbored a second E255K mutation (100%) and achieved CMR after olverembatinib treatment. Among them, the oldest patient was 69 years old (Liu et al., 2023). Similarly, olverembatinib was employed to treat six pediatric relapsed Ph^+^-ALL patients, and four of the five evaluable patients achieved CMR (Li et al., 2023). Inspired by these reports, in our report we treated a relapsed Ph^+^-ALL patient with olverembatinib monotherapy. The present patient was 79 years old, making her the oldest patient reported to date. Encouragingly, we observed CMR after two cycles of olverembatinib monotherapy. Furthermore, the safety profile was very good without dose reduction.

The prevention of CNS-L is of particular concern in Ph^+^-ALL treatment (Paul et al., 2023). According to the National Comprehensive Cancer Network (NCCN) guidelines for Ph^+^-ALL, CNS prophylaxis remains part of the standard care regardless of BCR/ABL1 TKI application. A study *in vitro* revealed that olverembatinib may have a potent ability to penetrate into the CNS, which strongly suggests the prophylactic role in CNS-L (Xiang et al., 2023). Thus, it may be possible to spare preventive intrathecal injections for CNS-L in clinical settings of third-generation BCR/ABL1 TKI. However, the clinical effect of the intrathecal injection–free regimen needs to be further validated in large-sample clinical studies.

## Conclusion

We described the case of an elderly patient who received oleverembatinib monotherapy for relapsed Ph^+^-ALL with E255V mutation. During the induction and maintenance treatment, CMR was observed for a further 6 months following oleverembatinib alone. At present, there is an ongoing paradigm shift in the use of chemotherapy-free regimens, possibly sparing the need for both intensive chemotherapy and allo-SCT. This case report describes the use of a novel therapeutic strategy of third-generation BCR/ABL1 TKI monotherapy in a fragile elderly patient with relapsed Ph^+^-ALL. Of course, the long-term efficacy of the treatment remains to be verified with further observation. With the advent of novel agents including blinatumomab, there is hope that Ph^+^-ALL will shift from a deadly leukemia to one that is largely curable.

## Data Availability

The original contributions presented in the study are included in the article/Supplementary material, further inquiries can be directed to the corresponding author.
